# Influence of Family Health History and Parental Education on Body Mass Index, Blood Pressure, and Lipid Levels in 5th Grade Children: A Secondary Data Analysis of the CARDIAC Project

**DOI:** 10.13023/jah.0704.02

**Published:** 2025-12-01

**Authors:** Madha Rani Das, Lee A. Plyes, Eloise Elliott, Christa L. Lilly

**Affiliations:** West Virginia University; West Virginia University; West Virginia University; West Virginia University

**Keywords:** Appalachia, blood pressure, BMI, CARDIAC Project, cardiovascular risk, childhood obesity, family history, lipid profiles, parental education

## Abstract

**Introduction:**

Childhood obesity and related cardiovascular risks reflect the interplay of genetic predispositions and socioeconomic adversity. Appalachia’s poverty, limited health care, and educational gaps worsen family health risks, driving early cardio-metabolic problems in children, highlighting the need for effective interventions.

**Purpose:**

The purpose of this study is to examine the association of family health history and parental education with BMI, BP, and lipid profiles among 5^th^ grade children in West Virginia (WV).

**Methods:**

The study used WV CARDIAC data from 2006–2009. Outcomes were BMI, SBP/DBP, and fasting lipids (HDL, LDL, TG). Predictors were parental education and family health history (heart disease, diabetes, high cholesterol, smoking). Multiple linear regression was used, adjusting for covariates.

**Results:**

Data from 30,756 fifth-grade children were analyzed. Among participating children, positive family history of smoking, diabetes, and high cholesterol was significantly associated with higher BMI (β = 3.07, 95% CI: 2.15–3.99, *p* < 0.0001). Paternal education showed a protective effect, with children of college-graduated fathers having lower BMI (β = −3.40, 95% CI: −4.20 to −2.60, *p* < 0.0001) and lower SBP (β = −0.94, 95% CI: −1.38 to −0.50, *p* < 0.0001) compared with those whose fathers had only a high school or GED education. Smoking (β = −0.69, 95% CI: −0.94 to −0.43, *p* < 0.0001) and diabetes (β = −0.48, 95% CI: −0.71 to −0.26, *p* < 0.0001) exhibited lower HDL levels.

**Implications:**

These findings highlight the effects of familial factors in shaping pediatric cardiovascular risk and emphasize the need for culturally tailored, family-centered interventions to reduce health disparities.

## INTRODUCTION

Obesity is a persistent and intricate condition influenced by a variety of contributing factors,^1^ and obesity in childhood has emerged as a significant and growing public health concern worldwide.^2^ Failure to recognize underlying predictors of this condition such as inherited predispositions and socioeconomic factors early in life can worsen the risk, affecting not only the individual child but also society as a whole. This is particularly true in the United States (U.S.), where obesity has reached epidemic proportions, contributing to over 335,000 deaths and 11.6 million disability adjusted life-years annually, driving healthcare costs beyond $260 billion. Childhood obesity affects nearly 20% of U.S. youth and increases the risk of early diabetes, blood pressure and adverse lipid profiles that foreshadow cardiovascular disease (CVD).^3^ Compounding this burden, the Appalachian Region in the U.S. is characterized by higher poverty, limited access to healthy foods, and lower educational attainment. Obesity rates in the region are considerably higher than national averages. For instance, a 2014 study of Southern Appalachian Adolescents found a 26.6% obesity rate^4^ (v national average ~20%),^5^ and prevalence of obesity in Appalachian counties has been reported at ~31% compared to ~27% in non-Appalachian U.S. counties.^6^ These disproportionately high rates of obesity in Appalachia are especially concerning given the well-established links between obesity and adverse cardiometabolic outcomes. Various indicators are related to early-life obesity, such as elevated blood pressure, abnormal lipid levels, and atherosclerosis,^7^ which heighten the risk of developing CVD and related complications later in life.^8^ Longitudinal evidence from U.S. Bogalusa Heart Study shows that children with high low-density lipoprotein Cholesterol (LDL-C) (≥130 mg/dL) had a markedly greater risk of adult dyslipidemia including ~5.4-fold higher high LDL along with higher obesity and hypertension 15 years later.^9^

Understanding the determinants of these health outcomes in children is critical for developing effective prevention and intervention strategies. A dynamic interplay between genetic susceptibility and environmental factors generally drives the initiation and evolution of obesity.^10^ A case-control study reported a significantly higher prevalence of dyslipidemia among children of ischemic heart disease (IHD) patients compared to their age-matched peers.^11^ Familial hyperlipidemia and familial combined hyperlipidemia are inherited conditions caused by LDL receptor mutations that lead to high LDL cholesterol levels.^12^ Since a family history of heart disease is strongly linked to high cholesterol in both children and adults,^13^ the American Academy of Pediatrics (AAP) and the National Heart, Lung and Blood Institute (NHLBI) issued integrated cardiovascular health guidelines in 2011, recommending universal lipid screening at ages 9–11 and 17–21, with earlier selective screening from age 2 for children with a family history of premature cardiovascular disease or elevated parental cholesterol.^14^ In terms of familial history, an analysis conducted on the Framingham Heart Study involving 6,278 parent–child trios (two parents and child) found that parental history of CVD, obesity, and smoking significantly increased the risk of future CVD in offspring, with parental obesity and smoking showing hazard ratios of 1.32 and 1.34, respectively.^15^ Another study conducted in Italy revealed a significant association between a family history of diabetes and various measures of childhood obesity, including children’s body mass index (BMI), subscapular area muscle, and waist circumference (WC).^16^

### Social Determinants of Childhood Obesity and Cardiovascular Health

Obesity in childhood and its related impact on cardiovascular health, conceptualized as the attainment of optimal levels across the American Heart Association’s Life’s Essential 8,^17^ is also significantly influenced by various social determinants, such as socioeconomic factors, parental education, and socio-demographic characteristics.^18^,^19^ However, the level of parental education can directly or indirectly influence parenting practices, children’s food choices or dietary habits, and shape their overall health outcomes. Parents are usually the first to teach children about nutrition, as their feeding practices, beliefs, and attitudes toward food shape the foundation of their children’s eating habits for the long term.^20^ Several studies have demonstrated the impact of parental socio-economic status (SES) indicators on childhood obesity. In the U.S., a study using the National Survey of Children’s Health reported that children of less-educated parents had substantially higher odds of obesity than those whose parents held a college degree (PR= 0.052, 95% CI:0.37–0.75), with lower household income further amplifying the risk.^21^ Another analysis of the U.S. 2007 National Survey of Children’s Health reported persistent and highly variable disparities in childhood obesity and pronounced socioeconomic gradients with higher prevalence among children from lower-education and lower income households.^22^ Consistent with this gradient, a study using nationally representative Add Health data showed that higher parental education and family income related to lower prevalence of overweight, particularly among white females.^23^ Comparable links have also been observed internationally; for instance, in Malaysia, a study explored the relationship between socioeconomic status and obesity in 12-year-old adolescents and found significant relationships between BMI categories in urban school adolescents with both maternal and paternal education.^24^ A similar pattern is evident in another research study conducted on six-year-old children in Germany that revealed a strong dose-response relationship between social class and obesity, showing that children belonging to the lowest social strata were more than three times as likely to be obese, compared to those from the highest social strata. Additionally, the study found that parental education was the most influential factor associated with childhood obesity.^25^ Also a systematic review examining 20 studies identified parental education, including maternal (n = 12), paternal (n = 6), and combined parental education (n = 8), as SES indicators, with 15 of these studies reporting an association between lower parental education and child adiposity.^26^

### Appalachia

West Virginia (WV) is the only U.S. state located entirely in the Appalachian Region. Nearly 50% of WV children are overweight or obese, with many showing signs of metabolic syndrome but lacking prior diagnosis or weight management advice.^27^

The Coronary Artery Risk Detection in Appalachian Communities (CARDIAC) project was initiated in 1998 as a school-based screening in three rural WV counties; it later expanded to all 55 counties and more than 480 schools with the aim of reducing CVD rates in WV.^28^–^31^ The initiative of the WV CARDIAC Project is crucial because it focuses on helping children in Appalachia who have limited access to care and come from low-income families where obesity and associated health problems are widespread issues.^8^

### Summary

Although family history of various health conditions and socioeconomic factors are well-established predictors of childhood obesity and cardiometabolic risk, they are often studied separately. Examining their independent contribution within the same population provides a clear picture of how inherited health risks and parental education levels are associated with early life health. However, a few studies have explored the effects of these factors on childhood health outcomes. Therefore, this study seeks to examine the association of family health history (including heart disease, smoking, diabetes, and cholesterol) and parental education level with BMI, blood pressure, and lipid levels in fifth-grade children. Drawing on prior CARDIAC publications and the literature, we hypothesize that family health history and parental education level is significantly associated with BMI, blood pressure, and lipid levels in fifth-grade children

## METHODS

This study utilized de-identified data obtained from the WV CARDIAC Project database, which is a large-scale screening program for 5^th^ grade students in WV. Based on the study aim and the variables of interest, four years of data from 2006 – 2009 were included in this secondary analysis to examine the association of family history and parental education level with BMI, blood pressure, and lipid levels. This data included a total of 30,756 WV children’s information about anthropometric, BP, lipid profiles, parental education, family health history, sex, age, and race.

### Ethics Approval

The Institutional Review Board (IRB) at West Virginia University (WVU) provided ethical approval, ensuring compliance with human subject protection guidelines, of a secondary data analysis of the CARDIAC dataset (IRB protocol number 2502118256). The initial WV CARDIAC Project also underwent IRB approval. More details can be found in Elliott et al., 2017.^25^ Prior to conducting any assessments, written authorization was secured from the superintendents of all 55 participating county schools; active consent from parents and assent from the participating children were obtained.^8^,^28^,^32^ All eligible children received a consent packet for parental review before participation. The packet included a detailed explanation of the study’s purpose, screening procedures, reporting methods, potential risks, and benefits.^33^ Parents who provided consent also completed a demographic form, which collected information such as family history of heart disease and diabetes, child’s name, address, and gender.^8^,^34^

### Assessment and Measures

Screenings were conducted in designated school areas by trained healthcare professionals, health science students, and volunteer phlebotomists. All health science students participating in screenings were trained through WV CARDIAC staff, site coordinators, or preceptors, ensuring accurate and reliable measurement procedures.^8^ Standardized procedures were used to measure anthropometric and cardiovascular parameters. The SECA Road Rod Stadiometer and SECA 840 Personal Digital Scale were used to measure children’s height and weight after students removed shoes and outerwear. BMI percentiles were calculated using Centers for Disease Control and Prevention (CDC) Epi Info V.3.5.4 software, based on age-and sex-specific CDC growth charts. Resting systolic (SBP) and diastolic (DBP) blood pressure were measured after a five-minute rest, using the first Korotkoff sound for SBP and the fifth Korotkoff sound for DBP. Fasting lipid profiles (FLP) were obtained, including total cholesterol (TC), high-density lipoprotein cholesterol (HDL), low-density lipoprotein cholesterol (LDL), and triglycerides (TRIG). HDL were subtracted from TC to calculate non-HDL cholesterol^8^,^28^,^35^ per NHLBI and AAP lipid guidance. Non-HDL-C is the cholesterol content of all atherogenic lipoproteins (including VLDL, LDL, IDL and lipoprotein a).^36^

The CARDIAC parental demographic survey questionnaire documented the presence of premature coronary heart disease (CHD), hypertension, diabetes, stroke, and high cholesterol (>240 mg/dl) among parents and grandparents. Presence of family history of premature CHD was defined as any of the following occurring in a parent or grandparent before the age of 55 years (the age of premature heart disease for men): myocardial infarction, angina requiring nitroglycerin therapy, coronary artery bypass surgery, balloon angioplasty, or sudden cardiac death. In addition to family history, the questionnaire also gathered information on parental education level and smoke exposure.^37^ Parental education information was treated as a nominal variable, combined and categorized into three levels: “Less than high school” (including parents who completed 8^th^ grade or less and some high school), “High school education or equivalent” (including parents who completed high school or General Educational Development (GED) and some college or technical training), and “College graduate or graduate school” (including parents who completed college and graduate school). The “high school education or equivalent” was set as the referent group in the analysis. Family history of cardiovascular risk factors, including heart disease, smoking, diabetes, and high cholesterol, were initially recorded as categorical variables (“at risk” or “not at risk”) based on the presence of the condition.

To facilitate statistical analysis, these variables were recoded into binary numeric values, where “at risk” was coded as 1 and “not at risk” as 0.

### Statistical Analysis

All the analyses were conducted by using JMP® Pro 17.0.0. (2022 JMP Statistical Discovery LLC, Carry, North Carolina, U.S).^38^ Statistical significance for all analyses was determined by an alpha of 0.05 unless otherwise specified. Missing data were addressed using pairwise deletion. To determine pairwise deletion appropriateness, we explored missing data patterns on the six outcomes variables. The presence or absence of missing data were recoded and compared to available demographic variables using spearman non-parametric correlations. Correlations were generally small (<0.2), suggesting missing completely at random data.

Descriptive analyses included the calculation of means and standard deviations for continuous variables and frequencies and valid percentages for categorical variables.

To examine the association of familial history (heart disease, smoking, diabetes, and cholesterol) and parental education level (mother’s and father’s education) with BMI percentile, blood pressure (systolic and diastolic), and lipid panel measures (HDL, LDL, log-transformed TG), we fit six multivariable linear regression models, for each of the six outcomes. The outcome variables comprised BMI percentile, SBP, DBP, and the lipid panel components (HDL, LDL, and TG). Ordinary Least Squares (OLS) regression models used standard selection techniques (that is, all variables entered simultaneously) and pairwise deletion for missing data. Model assumptions, including normality, homoscedasticity, and independence of residuals, were checked to ensure the validity of the results. Normality of residuals was violated for the log-normal variable of triglycerides, so triglyceride values were log transformed prior to analysis. Multicollinearity among predictors was assessed using variance inflation factors with a VIF >5 considered concerning; this assumption was not violated. All other assumptions were found satisfactory. The regression models fit effect sizes were evaluated using both R-squared and adjusted R-squared values. In addition, beta weight estimates with accompanying *p*-values for each predictor were reported. Covariates such as the child demographics of sex, age, and race were accounted for in the analysis to control for potential confounding effects.

## RESULTS

### Descriptive Analysis

A total of 30,756 children were included in this analysis. Table 1 shows the descriptive information of the participants. The sample consisted of 53.4% females and 46.6% males. The majority of participants were white (92.8%), and 7.2% were identified as a race other than white, with an average age of 11 years. The most common paternal and maternal education level was high school graduate or GED, or some college (65.4% fathers, 61.5% mothers). Positive family history of heart disease was reported in 30.9% of children, smoking exposure in 33.2%, diabetes in 50.1%, and high cholesterol in 36.7%.

Children’s mean BMI percentile was 17.7 (SD = 22.82). The mean SBP was 108.57 mmHg (SD = 11.99), DBP was 68.41 mmHg (SD = 9.31). Mean HDL of 50.39 mg/dl (SD = 12.11), LDL of 92.00 mg/dl (SD = 25.25), and TG had a mean value of 159.5 mg/dl (SD = 28).

### Inferential Analysis

Multivariable OLS linear regression models were used to assess the association of parental education level and family history of heart disease, smoking, diabetes, and cholesterol with participant BMI percentile, SBP, DBP, HDL, LDL, and log transformed TG.

### BMI Percentile

Table 2 shows the model examining predictors of BMI percentile. The overall model was statistically significant, with an overall F-ratio of 34.41 (*p* < 0.0001*), explaining approximately 3% of the variance in BMI percentile (R^2^ = 0.03, Adjusted R^2^ = 0.03). After adjusting for age, gender, and race, several family history and parental education factors were significantly associated with children’s BMI percentile. Children who have a family history of smoking had significantly higher BMI percentiles compared to those without, with an estimated increase of β = 1.32, *p* < 0.0001. Similarly, a family history of diabetes and high cholesterol were associated with increased BMI percentiles of β = 1.59, *p* < 0.0001, and β = 1.36, *p* < 0.0001, respectively. Parental education, particularly paternal education, demonstrated a strong association with BMI percentile. Compared to children whose fathers completed high school or went to college (reference group), those whose fathers had not completed high school or had an education level of 8th grade or less had significantly higher BMI percentiles (β = 3.07, *p* < 0.0001*). Moreover, children whose fathers were college graduates had 3.40 points lower BMI than the reference group (β = −3.40, *p* < 0.0001*). Maternal education was shown no significant association with BMI percentile in this model.

### Systolic Blood Pressure (SBP)

A statistically significant result (*p* < 0.0001*) was found for the overall SBP model with an overall F-ratio of 18.09, explaining approximately 2% of the variance in SBP (R^2^ = 0.02, Adjusted R^2^ = 0.02).

Estimates can be found in Table 3. Children who have a family history of heart disease (β = 0.43, *p* = 0.0007), and those with a family history of diabetes (β = 0.34, *p* = 0.0042) and high cholesterol (β = 0.56, *p* < 0.0001) had significantly higher SBP than those without. For parental education, father’s education level showed a protective effect: children whose fathers were college graduates or in graduate school had SBP levels 0.94 mmHg lower (β = −0.94, *p* = <.0001*) compared to those whose fathers completed high school or went to college (reference group). Maternal education was not significantly associated with SBP in this model.

### Diastolic Blood Pressure (DBP)

Table 4 shows the results for DBP. The analysis of DBP followed a similar pattern. A statistically significant result with an F-ratio of 10.47, explaining 1% of the variance in DBP (R^2^ = 0.01, Adjusted R^2^ = 0.01) was found. Children with a family history of heart disease (β = 0.25, *p* = 0.0093*) and cholesterol (β = 0.37, *p* < 0.0001*) had higher DBP values. Additionally, higher levels of paternal education were associated with lower DBP in children, though the effect was less pronounced compared to SBP.

### High Density Lipoprotein Level (HDL)

A statistically significant result was also found for HDL cholesterol with an overall F-ratio of 24.84 (*p* < 0.0001*), explaining 2% of the variance in HDL levels (R^2^ = 0.02, Adjusted R^2^ = 0.02). Table 5 shows that several predictors, including family history and parental education, were significantly associated with children’s HDL levels. In the present analysis, HDL levels demonstrated a negative correlation with factors such as smoking and diabetes. Results indicated that children with a family history of diabetes and smoking had 0.48-and 0.69-point lower HDL level (β =−0.48, *p* =<.0001* and β =−0.69, *p* =<.0001* respectively), highlighting the detrimental impact of these lifestyle factors on cardiovascular health. In terms of parental education, children whose fathers have a high school degree (reference group) have higher HDL compared to those have only 8^th^ grade or less. Similarly, children whose fathers completed a college degree or had some post-graduate education tend to have higher HDL levels compared to those whose fathers have only completed high school (reference group). The positive β estimate (0.98) suggests a similar trend, with higher paternal education being associated with higher HDL levels in children. Mother’s education level, however, does not show any significant effect on HDL levels in children in this study.

### Low Density Lipoprotein Level (LDL)

The analysis of LDL also yielded a significant result, *p* =<0.0001*, and the model explaining 2% variance in LDL results, as shown in Table 6. The results showed that children with a family history of heart disease had higher LDL levels with an estimated increase of β = 0.82, *p* = 0.0015. Additionally, a family history of abnormal cholesterol was significantly associated with elevated LDL levels (β =3.19, *p* =<0.0001*). However, a history of diabetes was associated with lower LDL levels (β =−0.495, *p* =<0.0379*). For parental education level, the father’s education demonstrated a weaker, but notable association with LDL levels. Children whose fathers are college graduates tend to have lower LDL levels compared to children whose fathers only graduated from high school (Reference group), though the result was not significant.

### Log Transformed Triglycerides (TG)

The analysis of log transformed TG revealed a statistically significant result with an F-ratio of 34.33 (*p* < 0.0001*), explaining 3% of the variance in triglyceride levels (R^2^ = 0.03, Adjusted R^2^ = 0.03). Triglyceride value is log transformed due to higher variability and non-normality of range of levels. Estimates are presented in Table 7. The β estimate = −0.05 with a *p*-value = <0.0001* indicates a negative relationship between father’s education (College graduate or graduate school compared to High school GED or college) and Log TG levels. This suggests that children whose fathers are college graduates or in graduate school tend to have slightly lower Log TG levels compared to children whose fathers have only completed High school GED or in college. The 95% Confidence Interval for the β estimate is −0.07 to −0.03, indicating that this effect is statistically significant. On the other hand, the β estimate = 0.05, with a *p*-value = <0.0001*, suggests that children whose fathers have completed ≤8^th^ grade or some high school tend to have slightly higher Log TG levels than those whose fathers are high school graduates (Reference group). The 95% Confidence Interval for this estimate is 0.03 to 0.07, which is also statistically significant. Similarly, children whose mothers completed ≤8^th^ grade or some high school compared to high school graduates tend to have slightly higher Log TG levels (β = 0.03, *p* = 0.0168*). Also, children whose mothers are college graduates or in graduate school tend to have slightly lower Log TG levels compared to children whose mothers have only completed high school or GED or in college (β =−0.02, *p* =0.0127*). In terms of family history of health conditions, smoking and diabetes showed a weak positive relation with Log TG levels (β =0.02, *p* =0.0002* and β =0.01, *p* =0.0998 respectively). A strong positive association between family history of cholesterol and Log TG was found, indicating that children with a family history of high cholesterol have significantly higher triglyceride levels (β =0.04, *p* =0.0001*).

## DISCUSSION

This secondary data analysis aimed to investigate the association of family history and parental education level with BMI percentile, blood pressure, and lipid profiles in a large sample of fifth-grade children from the WV CARDIAC Project. The findings of this secondary data analysis offer compelling evidence on how intergenerational health risks and socioeconomic status shape early cardiometabolic profiles and are particularly salient in Appalachia, where higher poverty, limited access to healthy foods and education, and disproportionately elevated obesity rates compared with national averages amplify children’s vulnerability.

Our results demonstrate that a family history of smoking, diabetes, and high cholesterol is significantly associated with higher BMI percentiles in children. These results support previous research and highlight the interplay between inherited risk and environmental exposure in the development of childhood obesity and metabolic dysregulation.^11^,^15^,^16^ These studies have shown that children with a family history of CVD or diabetes exhibit significantly higher BMI, dyslipidemia, and altered lipid profiles, with parental obesity, smoking, and sedentary lifestyle further increasing the risk, emphasizing the need for early screening and targeted prevention strategies for children at higher risk due to family history and modifiable factors such as diet and physical activity. Although parental smoking is a behavioral risk factor rather a clinical health condition, it is included in the family health history variable as parental smoking is associated with offspring’s cardio-metabolic health. For example, prior study has been reported that exposure to secondhand smoke was associated with lower HDL-C and higher markers of atherogenic lipoproteins^39^ which supports this study’s results. Another study on a European cohort found that maternal smoking during pregnancy was associated with higher SBP percentile in children at age 11, showing genetical linkage between family history of smoking and health outcomes.^40^

Paternal education emerged as a consistent protective factor across multiple health outcomes, including BMI, SBP, HDL, and TG levels in this analysis. Compared to children of less-educated fathers, those with fathers who were college graduates had significantly healthier cardiometabolic indicators. This is consistent with earlier findings where parental education levels, particularly paternal, was inversely associated with childhood obesity and metabolic risk.^24^–^26^ These results reinforce the broader role of parental education as a proxy for or a driver of SES, access to health-related resources, and health literacy. Interestingly, maternal education did not show significant associations in most models, diverging from prior literature emphasizing its importance.^26^

Blood pressure findings further corroborate the importance of family health history. Children with familial history of heart disease, diabetes, and cholesterol exhibited elevated SBP and DBP. These results are in line with previous findings where studies have shown that childhood obesity significantly increases the risk of hypertension, with overweight or obese girls being two times more likely to have elevated SBP indicative of pre-hypertension or hypertension, and obesity in children is associated with a threefold higher risk of hypertension, highlighting the importance of early prevention and long-term management of obesity and its cardiovascular complications.^41^,^42^

In terms of lipid profiles, children with a family history of heart disease, high cholesterol, and diabetes had lower HDL levels, an unfavorable pattern associated with increased CVD risk. On the other hand, higher LDL levels were observed among children with a family history of heart disease, high cholesterol and diabetes. Additionally, elevated TG levels were associated with parental smoking and high cholesterol. The results of this study, although unique in their direct association between family history of heart disease, diabetes, smoking, and lipid profiles in children, are consistent with clinical guidelines. According to the Lipid Screening in Children and Adolescents guidelines by Nationwide Children’s Hospital,^43^ children with a family history of early CVD, diabetes, or tobacco use are recommended for selective lipid screening. However, the American Academy of Pediatrics Guideline for lipid screening advocates universal cholesterol screening at ages 11 and 18 years, and this was promulgated in large part based on previous findings of abnormal lipid levels from WV CARCIAC Project in the absence of family history of heart disease.^34^ Thirty percent of children with LDL-C above 130 mg/dl had negative family history for coronary heart disease. The current findings suggest that a family history of diabetes and smoking may be associated with lipid abnormalities in children, warranting early screening and intervention. Again, paternal education levels showed a beneficial relationship with HDL and TG levels, as higher parental education was associated with higher HDL and lower TG levels, reinforcing the socioeconomic gradient in pediatric cardiovascular health. However, LDL levels were not significantly associated with parental education in this cohort as LDL is often more strongly driven by genetic or longer-term cumulative exposures.

Although this study focuses on Appalachian children, the relationships between parental education and family health history on children cardiometabolic outcomes reflect broader social and familial mechanisms that have been documented in non-Appalachian populations. The findings are therefore likely applicable beyond Appalachia especially in similarly disadvantaged communities while the concentration of poverty and limited resources in this region may intensify the observed gradients. Although the data analyzed in this study are from 2006–2009, the burden of obesity, hypertension and dyslipidemia among U.S. children remains high. Nearly 20% of U.S. children now have obesity,^44^ and pediatric hypertension affects an estimated 2–4%.^45^ Childhood obesity, hypertension and dyslipidemia continue to disproportionately affect Appalachian children, with family history and parental education persisting as key determinants of risk. By demonstrating how these factors are associated with BMI, blood pressure, and lipid levels, this study’s findings inform ongoing screening, prevention, and intervention efforts that address both intergenerational and socioeconomic factors in pediatric CVD health.

### Strengths and Limitations

Taken together, these findings emphasize that cardiometabolic risk in children is not solely a consequence of personal behavior but is heavily shaped by family background, both genetic and socioeconomic. These patterns are particularly important in underserved rural populations like Appalachia, where health disparities are exacerbated by limited access to health care, lower than average education levels, and entrenched poverty.^32^,^46^ A recent report from the Appalachian Regional Commission suggests health is improving in Central Appalachia but at a rate less than the remainder of the country; in fact, Central Appalachian health disparity is increasing.^47^ Methodologically, a major strength of this study is the objective assessment of most variables using standardized protocol including measured height and weight, resting SBP and DBP and fasting lipid panels by trained personnel.

While this study offers valuable insights, it also has limitations. The use of secondary data from the CARDIAC Project (2006–2009), while beneficial due to its large, well-structured dataset, limits the study to the research questions and variables included in the original design. However, secondary data analysis offers the advantage of working with a substantial dataset without the constraints of primary data collection, allowing for the exploration of additional research questions that were not part of the original study. Due to the study design, there were significant variations in missing data on all variables which can lead to bias under missing at random or missing not at random data when using pairwise deletion. It can also limit comparability of results across outcomes. However, it appears that missing data on outcomes have weak associations to available demographic variables, suggesting missing completely at random data. Also, pairwise deletion preserves more information while still providing unbiased estimates under MCAR assumptions, which applied to this study data. Another limitation is the use of an older dataset (2006–2009), which may limit current relevance of prevalence estimates; however, because the study focuses on associations rather than time-specific prevalence, the results remain translatable to current day and help identify predictors for early screening.

## CONCLUSION

This study demonstrates that both family history of several health conditions and parental education level significantly associated with BMI, blood pressure, and lipid profiles among fifth-grade children in WV. The findings from the analysis suggest that HDL, LDL, and TG levels are significantly influenced by a combination of demographic factors, lifestyle choices, and health conditions. Particularly, family history of smoking, diabetes, and elevated cholesterol levels were identified as key factors that adversely affect children’s lipid profiles, thereby increasing the likelihood of cardiovascular complications. These findings underscore the need for early, family-centered interventions, particularly in regions with persistent socioeconomic disadvantages.

The results also reinforce the value of school-based screening programs like the WV CARDIAC Project, which can identify at-risk children before the onset of clinical disease. Future public health strategies should incorporate both genetic risk assessment and socioeconomic support—especially educational resources for parents—to mitigate the intergenerational transmission of cardiovascular risk.

Efforts to reduce childhood obesity and improve cardiovascular health must therefore consider not only biological inheritance but also social determinants of health. Multilevel approaches that include parental education, household behaviors, and structural inequities are vital for creating lasting improvements in children health outcomes, particularly in medically underserved areas such as rural Appalachia.

**SUMMARY BOX**

**What is already known about this topic?**

Childhood overweight and obesity are the most common nutritional problems in the U.S. and significantly increase the risk of developing additional CVD risk factors during childhood and adolescence. A complex interplay of genetic predisposition, socioeconomic status, and parental education contribute substantially to the risk and development of obesity in children. Lifestyle factors, including poor diet quality, physical inactivity, and sedentary behaviors, are also well-established contributors. Furthermore, obesity prevalence shows disparities across racial, ethnic, and socioeconomic groups. Despite numerous interventions, sustaining long-term weight management remains challenging, highlighting the importance of early prevention efforts.

**What is added by this report?**

This study uniquely examines the association of family health history and parental education with BMI, blood pressure, and lipid profiles in a large cohort of Appalachian 5^th^ grade children. Results highlights that paternal education, more so than maternal education, has a protective effect on children’s cardiometabolic health. The study shows the links between specific familial health conditions and increased cardiometabolic risk, underscoring the complex nature of childhood obesity.

**What are the implications for future research?**

Future research should develop and evaluate family-centered, culturally tailored interventions addressing both genetic and socioeconomic factors to reduce pediatric cardiovascular risk in Appalachia. Longitudinal studies are needed to track how familial and socioeconomic factors interact over time to influence cardiometabolic outcomes. This will offer a clear foundation for public health strategies for preventing cardiometabolic diseases, especially for populations at higher risk due to their family or socioeconomic background.

## Figures and Tables

**Table 1:** Demographics of Coronary Artery Risk Detection in Appalachian Communities Fifth-Grade Participants (N = 30,756)

Variable	Category/Unit	N (Valid %) or Mean (SD)
**Child Demographics**		
*Sex*		
	Female	16,428 (53.4%)
	Male	14,328 (46.6%)
*Race*		
	White	24,275 (92.8%)
	All other	1,897(7.2%)
*Age (n=30,654)*		
	Years	11.00 (0.51)
**Parent Education**		
*Father’s Education*		
	Less than high school	3,083(13.7%)
	High school graduate, GED, or some college	14,673(65.4%)
	College graduate or graduate school	4,694(20.9%)
*Mother’s Education*		
	Less than high school	2,124(8.9%)
	High school graduate, GED, or some college	14,600(61.5%)
	College graduate or graduate school	6,985(29.4%)
**Family History**		
*Heart Disease*		
	Yes	5,495 (30.9%)
	No	12,291 (69.1%)
*Smoke Exposure*		
	Yes	8,369 (33.2%)
	No	16,811 (66.7%)
*Diabetes*		
	Yes	12,197 (50.1%)
	No	12,167 (49.9%)
*High Cholesterol*		
	Yes	7,919 (36.7%)
	No	13,648 (63.2%)
**Children Outcomes: Anthropometric & Health Markers**		
*Systolic Blood Pressure (n=28,486)*		
	mmHg	108.57 (11.99)
*Diastolic Blood Pressure (n=28,467)*		
	mmHg	68.41 (9.31)
*High Density Lipoprotein Cholesterol (n=24,126)*		
	mg/dl	50.39 (12.11)
*Low Density Lipoprotein Cholesterol (n=24,087)*		
	mg/dl	92.00 (25.25)
*Triglycerides (n=24,240)*		
	mg/dl	159.5 (28)
*Body Mass Index Percentile (n=30,153)*		
	%	70.64 (28.91)

**Table 2:** Regression Model for the Influence of Parental Education and Family History on BMI Percentile in 5th Grade Children (N = 11,631)

Predictor	Category	β estimate	Standard error	t ratio	*p*-value	Lower 95% CI	Upper 95% CI
*Father’s education*							
	High school education or equivalent (Ref)						
	Less than high school	3.07	0.47	6.57	<.0001^*^	2.15	3.99
	College graduate or graduate school	−3.40	0.41	−8.33	<.0001^*^	−4.20	−2.60
*Mother’s education*							
	High school education or equivalent (Ref)						
	Less than high school	0.95	0.57	1.67	0.09	−0.16	2.07
	College graduate or graduate school	−0.96	0.41	−2.35	0.02	−1.75	−0.16
*Heart disease*							
	No (Ref)						
	Yes	0.48	0.23	2.09	0.0370^*^	0.03	0.94
*Smoke*							
	No (Ref)						
	Yes	1.32	0.24	5.42	<.0001^*^	0.84	1.79
*Diabetes*							
	No (Ref)						
	Yes	1.59	0.21	7.43	<.0001^*^	1.17	2.01
*Cholesterol*							
	No (Ref)						
	Yes	1.36	0.22	6.21	<.0001^*^	0.93	1.79
*Race*							
	White	−1.70	0.44	−3.92	<.0001^*^	−2.56	−0.85
	All Other (Ref)						
*Gender*							
	Female	−1.09	0.21	−5.21	<.0001^*^	−1.49	−0.68
	Male (Ref)						
*Age*							
	Years	−0.52	0.433	−1.21	0.23	−1.36	0.32

**Table 3:** Statistical Analysis of Parental Education and Family History as Predictors of SBP in 5th Grade Children (N = 11,631)

Predictor	Category	β estimate	Standard error	t ratio	*p*-value	Lower 95% CI	Upper 95% CI
*Father’s education*							
	High school education or equivalent (Ref)						
	Less than high school	0.78	0.26	3.05	0.0023	0.28	1.29
	College graduate or graduate school	−0.94	0.22	−4.19	<.0001^*^	−1.38	−0.49
*Mother’s education*							
	High school education or equivalent (Ref)						
	Less than high school	0.33	0.31	1.04	0.297	−0.29	0.94
	College graduate or graduate school	−0.25	0.22	−1.1	0.2719	−0.68	0.19
*Heart disease*							
	No (Ref)						
	Yes	0.43	0.13	3.39	0.0007^*^	0.18	0.68
*Smoke*							
	No (Ref)						
	Yes	0.23	0.13	1.76	0.0786	−0.03	0.49
							
*Diabetes*							
	No (Ref)						
	Yes	0.34	0.12	2.86	0.0042^*^	0.11	0.57
*Cholesterol*							
	No (Ref)						
	Yes	0.56	0.12	4.63	<.0001^*^	0.32	0.79
*Race*							
	White	−0.19	0.24	−0.79	0.4303	−0.66	0.28
	All Other (Ref)						
*Gender*							
	Female	−0.39	0.11	−3.45	0.0006^*^	−0.62	−0.17
	Male (Ref)						
*Age*							
	Years	1.83	0.24	7.75	<.0001^*^	1.37	2.29

**Table 4:** Regression Findings on the Impact of Parental Education and Family History on DBP in 5th Grade Children (N = 11,631)

Predictor	Category	β estimate	Standard error	t ratio	*p*-value	Lower 95% CI	Upper 95% CI
*Father’s education*							
	High school education or equivalent (Ref)						
	Less than high school	0.33	0.19	1.7	0.0899	−0.05	0.72
	College graduate or graduate school	−0.3	0.17	−1.77	0.0774	−0.64	0.03
*Mother’s education*							
	High school education or equivalent (Ref)						
	Less than high school	0.04	0.24	0.18	0.8536	−0.43	0.51
	College graduate or graduate school	0.01	0.17	0.09	0.9308	−0.32	0.35
*Heart disease*							
	No (Ref)						
	Yes	0.25	0.09	2.6	0.0093^*^	0.06	0.45
*Smoke*							
	No (Ref)						
	Yes	0.02	0.1	0.24	0.8078	−0.18	0.23
*Diabetes*							
	No (Ref)						
	Yes	0.12	0.09	1.28	0.2011	−0.06	0.29
*Cholesterol*							
	No (Ref)						
	Yes	0.37	0.09	3.97	<.0001^*^	0.19	0.55
*Race*							
	White	−0.75	0.18	−4.08	<.0001^*^	−1.11	−0.39
	All Other (Ref)						
*Gender*							
	Female	−0.27	0.09	−3.12	0.0018^*^	−0.45	−0.10
	Male (Ref)						
*Age*							
	Years	1.16	0.18	6.39	<.0001^*^	0.80	1.51

**Table 5:** Associations Between Parental Education, Family History, and HDL Levels in 5th Grade Children (N = 11,631)

Predictor	Category	β estimate	Standard error	t ratio	*p*-value	Lower 95% CI	Upper 95% CI
*Father’s education*							
	High school education or equivalent (Ref)						
	Less than high school	−1.05	0.25	−4.17	<.0001^*^	−1.54	−0.55
	College graduate or graduate school	0.98	0.22	4.49	<.0001^*^	0.55	1.41
*Mother’s education*							
	High school education or equivalent (Ref)						
	Less than high school	−0.18	0.31	−0.59	0.5559	−0.78	0.42
	College graduate or graduate school	0.19	0.22	0.89	0.3745	−0.23	0.62
*Heart disease*							
	No (Ref)						
	Yes	−0.21	0.12	−1.71	0.0876	−0.46	0.03
*Smoke*							
	No (Ref)						
	Yes	−0.69	0.13	−5.27	<.0001^*^	−0.94	−0.43
*Diabetes*							
	No (Ref)						
	Yes	−0.48	0.11	−4.2	<.0001^*^	−0.71	−0.26
*Cholesterol*							
	No (Ref)						
	Yes	−0.18	0.12	−1.53	0.1271	−0.41	0.05
*Race*							
	White	−1.12	0.23	−4.79	<.0001^*^	−1.58	−0.66
	All Other (Ref)						
*Gender*							
	Female	−1.10	0.11	−9.84	<.0001^*^	−1.32	−0.88
	Male (Ref)						
*Age*							
	Years	−0.84	0.23	−3.63	0.0003^*^	−1.29	−0.39

**Table 6:** Effect of Parental Education and Family History on LDL Levels in 5th Grade Children (N = 11,631)

Predictor	Category	β estimate	Standard error	t ratio	*p*-value	Lower 95% CI	Upper 95% CI
*Father’s education*							
	High school education or equivalent (Ref)						
	Less than high school	−0.06	0.52	−0.11	0.9101	−1.08	0.96
	College graduate or graduate school	−0.25	0.45	−0.56	0.5768	−1.14	0.64
*Mother’s education*							
	High school education or equivalent (Ref)						
	Less than high school	−0.54	0.63	−0.85	0.3953	−1.78	0.7
	College graduate or graduate school	0.17	0.45	0.37	0.709	−0.72	1.06
*Heart disease*							
	No (Ref)						
	Yes	0.82	0.26	3.18	0.0015^*^	0.32	1.33
*Smoke*							
	No (Ref)						
	Yes	0.15	0.27	0.56	0.5764	−0.38	0.68
*Diabetes*							
	No (Ref)						
	Yes	−0.49	0.24	−2.08	0.0379^*^	−0.96	−0.03
*Cholesterol*							
	No (Ref)						
	Yes	3.19	0.24	13.03	<.0001^*^	2.71	3.67
*Race*							
	White	−0.58	0.49	−1.19	0.23	−1.53	0.37
	All Other (Ref)						
*Gender*							
	Female	−0.30	0.23	−1.33	0.1842	−0.76	0.15
	Male (Ref)						
*Age*							
	Years	−2.65	0.48	−5.54	<.0001^*^	−3.59	−1.72

**Table 7:** Associations Between Parental Education, Family History, and Log TG Levels in 5th Grade Children (N = 11,631)

Predictor	Category	β estimate	Standard error	t ratio	*p*-value	Lower 95% CI	Upper 95% CI
*Father’s education*							
	High school education or equivalent (Ref)						
	Less than high school	0.05	0.01	4.91	<.0001^*^	0.03	0.07
	College graduate or graduate school	−0.05	0.01	−5.18	<.0001^*^	−0.07	−0.03
*Mother’s education*							
	High school education or equivalent (Ref)						
	Less than high school	0.03	0.01	2.39	0.0168^*^	0.01	0.06
	College graduate or graduate school	−0.02	0.01	−2.49	0.0127^*^	−0.04	−0.005
*Heart disease*							
	No (Ref)						
	Yes	0.003	0.01	0.66	0.5071	−0.01	0.01
*Smoke*							
	No (Ref)						
	Yes	0.02	0.005	3.75	0.0002^*^	0.01	0.03
*Diabetes*							
	No (Ref)						
	Yes	0.01	0.005	1.65	0.0998	−0.002	0.02
*Cholesterol*							
	No (Ref)						
	Yes	0.04	0.005	8.49	<.0001^*^	0.03	0.05
*Race*							
	White	0.07	0.01	6.78	<.0001^*^	0.05	0.09
	All Other (Ref)						
*Gender*							
	Female	0.05	0.005	10.91	<.0001^*^	0.04	0.06
	Male (Ref)						
*Age*							
	Years	0.02	0.01	2.57	0.0103^*^	0.01	0.04
